# A social media intervention for communicating vaccine safety in low- and middle-income countries: protocol for a pilot study

**DOI:** 10.3389/fpubh.2023.1248949

**Published:** 2023-12-07

**Authors:** Lucie Marisa Bucci, Smaragda Lamprianou, Francesco Gesualdo, Alberto E. Tozzi, Tala Ghalayini, Isabelle Sahinovic, Shanthi Pal

**Affiliations:** ^1^Bucci-Hepworth Health Services, Pincourt, QC, Canada; ^2^World Health Organization, Geneva, Switzerland; ^3^Predictive and Preventive Research Unit, Bambino Gesù Children’s Hospital, Rome, Italy; ^4^Accenture Health and Public Service, London, United Kingdom

**Keywords:** COVID-19, vaccine safety, communication, social media, surveillance, LMIC

## Abstract

Vaccine safety is a concern that continues to drive hesitancy and refusal in populations in low-and-middle income countries (LMICs). Communicating about vaccine safety is a strategy that can successfully change personal and community perceptions and behaviors toward vaccination. The COVID-19 infodemic emergency with the rapid rollout of new vaccines and new technology, demonstrated the need for good and effective vaccine safety communication. The Vaccine Safety Net (VSN), a WHO-led global network of websites that provide reliable information on vaccine safety offers the ideal environment for gathering web and social media analytics for measuring impact of vaccine safety messages. Its members work with a wide range of populations, in different geographic locations and at many levels including national, regional, and local. We propose to undertake a pilot study to evaluate the feasibility of implementing COVID-19 vaccine safety communications with VSN members working in LMICs and to assess the impact of communications on public knowledge, attitudes, and perceptions.

## Introduction

Vaccines remain effective public health interventions for reducing significant morbidity and mortality caused by communicable diseases in populations globally (1, 2). Despite the success of vaccines, populations continue to be hesitant and either delay or refuse vaccines. There is a myriad of complex interrelated factors that contribute to vaccine refusal (3), but the most cited concern is vaccine safety (4). The risk of harm is considered a driver of refusal particularly in low- and middle-income countries (LMICs) (5).

The perception of risk vs. benefit plays a pivotal role in individual and community decision-making about vaccination (6–8). In our digital world, perceptions about vaccines are commonly shaped by religion, culture, and politics through influencers found in a range of networks that are simultaneously offline and online (e.g., social networks) (9). While these social networks have indeed increased population awareness, they have also facilitated the confluence and circulation of inaccurate and misleading information that may cause harm to population health (10). Vaccine safety communication, which entails the fostering of vaccine confidence through a range of strategies (i.e., collecting analytical data through social media listening tools, diagnosing and identifying concerns as they arise, creation of common messaging), is widely recognized by experts globally as an effective intervention for addressing population concerns and for filling information voids caused by the spread of misinformation (11).

Providing people with risk–benefit information in comprehensible ways is a strategy for reducing doubt in vaccines and building resilience against misinformation. The rejection of misinformation requires mindfulness to critically assess what is falsely presented as fact (12). Most misinformation appeals to negative emotions, which has been shown to reinforce false beliefs, or any state of doubt along the vaccine hesitancy spectrum (3) as defined by the World Health Organization (WHO) Strategic Advisory Group of Experts (SAGE) in 2015. Vaccine hesitancy is also very context-specific and variable across time, places, and vaccines (3).

The 3C’s of vaccine hesitancy (complacency, confidence, and convenience), a mainstay conceptual model in vaccine demand and acceptance research, is a useful model for identifying and evaluating factors that lead to personal or community unwillingness to be vaccinated (13). This model was recently expanded by the WHO Behavioral and Social Drivers of Vaccination (BeSD) Working Group to include two more categories: rational calculation and collective responsibility, which provide additional explanations for vaccination decision-making (7). The value of the model is that it helps frame vaccine hesitancy issues. It is also understood by SAGE that this model should continue to be expanded upon through new learnings, tools, and best practices.

The Vaccine Safety Net (VSN) was created by the World Health Organization (WHO) in 2003 to promote vaccine safety communication by facilitating access to trustworthy information on accredited websites who meet the WHO’s good information practices criteria (14, 15). The VSN network currently includes 104 websites in all WHO regions that adhere to the Global Advisory Committee on Vaccine Safety (GACVS) good information practices criteria. In fact, to become a VSN member, mandatory criteria in terms of the credibility of the website, the quality and quantity of the content (i.e., vaccine safety related information), as well as the design and accessibility of the website should be met. VSN membership is accountable for ensuring quality content online and facilitating the access of internet users to science-based and reliable vaccine safety information. Members benefit from guidance on how to optimize search engine rankings. They also have access to tips for best practices in vaccine safety communication, how to improve web linking and web analytics gathering (16) to facilitate public access to evidence-based and trustworthy information through collaborations, webinars and projects organized by the WHO. Additionally, since its creation, the network has become a knowledge base for all members seeking to achieve more effective ways to communicate about vaccine safety. VSN-led research expanded to meet this need through web and social media analytics gathering practices within the network to measure the reach of the members’ communication efforts, and impact on population behavior. WHO contracted a social listening platform that supported VSN members to evaluate the outreach of their messaging and communication strategies through their respective social media accounts by monitoring particular parameters such as numbers of shares, likes, trends, retweets, comments, replies, and sentiments using their tools. The messages were used in local contexts such as vaccination campaigns, or during global events such as World Immunization Week (WIW).

The COVID-19 infodemic emergency demonstrated the need for effective and good communication because of the rapid global rollout of new vaccines and new technologies intended for different groups of users (in terms of age and underlining health factors such as chronic disease). The VSN provides an ideal environment for gathering unique web and social media analytics for measuring the impact of vaccine safety communications. The VSN is unique in terms of its richness of knowledge and expertise ranging from epidemiologists, clinical pharmacists, pediatricians, nurses, midwives, IT specialists and others. In addition, members come from different settings and often experience different cultural or public health contexts and issues. Its members work at many levels including national, regional, and local. Since the height of the COVID-19 pandemic, VSN members have reported diverging experiences with vaccine acceptance, confidence and even the spread of misinformation, which has posed challenges never experienced before. There has been pressing concern to address the continuous onslaught of information, ever changing, from a multitude of untrusted sources. In addition, circulating conspiracies about the side effects of COVID-19 vaccines have affected public confidence in vaccination (17). This is not a new issue; many recent immunization programs have suffered setbacks from misinformation and inadequate communication, such as the “HPV scare in Japan” (18–20).

The primary objective of this protocol is to call for the running of a pilot study with VSN members working in LMICs, where COVID-19 vaccine safety messaging customized to local settings will be disseminated through web and social media platforms. The second objective is to evaluate the feasibility of the pilot study by gathering feedback from VSN members. This is the first pilot study of its kind to be implemented in collaboration with VSN members from LMICs. To our knowledge, there are only a few projects comparable to this pilot study (21).

## Methods and analysis

**Figure 1:**
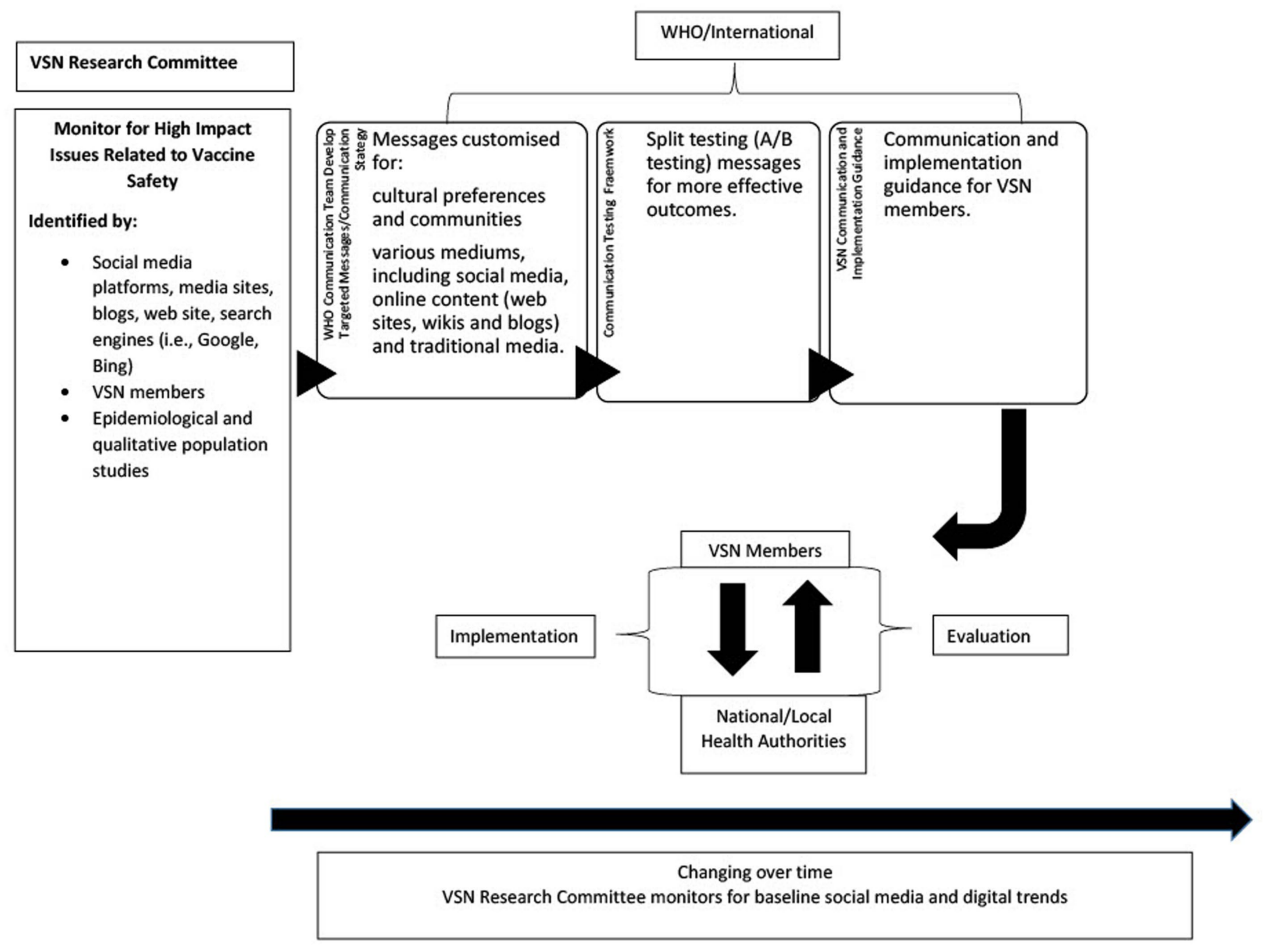
VSN social media framework.

The pilot study will be implemented in four phases: (1) intelligence gathering; (2) vaccine safety message development; (3) implementation and monitoring; and (4) evaluation. This comprehensive framework is depicted in Figure 1.

### VSN recruitment

A call for participation will be sent to the Network. Eligible members will be VSN members coming from LMICs with limited resources or members located in countries with very low vaccine coverage. All other members will be excluded from the study, but they can participate in the “Research Committee” that will provide support in terms of data analysis, social media intelligence, communication expertise, statistical analysis and social behavior analysis. The Research Committee will be composed of VSN members participating and not participating in the study, WHO representatives, a representative from the social listening platform and other stakeholders as needed.

### Intelligence gathering

A triangulation approach (22) will be used for collecting data that will be used to understand the epidemiological, socio-cultural, religious, and political views that would affect vaccine confidence. VSN members participating in the pilot study will collect baseline intelligence in their respective countries and settings. The study team will use multiple sources of information such as local media, disease surveillance, post-marketing pharmacovigilance reports, websites, and social media platforms to capture public perceptions. Data gathering using triangulation is a recognized approach in health research monitoring and evaluation (23) to understanding local contexts, improves credibility and validity of findings, and is therefore appropriate for this study.

In addition to the data collected in their settings, VSN members will be asked to respond to a short questionnaire that will prompt intelligence sharing on populations at high risk of severe disease and negative health outcomes. These will provide a context that will help to understand the setting-specific events. They will include items concerning:

- *Traditionally vaccine hesitant and under immunized communities*; understanding the drivers of hesitancy often translates to understand the concerns of people regarding the safety of vaccines.
- *Age-specific and chronically ill behaviors toward vaccination*.
- *Active community leaders/influencers*; understanding vaccination perception of community leaders and influencers will inform on the community sentiment toward vaccination and immunization campaigns. In addition, establishing relations and involving influential people of the community in the survey, will support responsiveness.
- *Events that impact public perception of vaccine safety*; particular events that occurred in a setting might influence people’s perception about vaccines. A poorly managed event will erode trust of the community for immunization programs, public health services and in general vaccination.
- *Research information* (i.e.*, country specific research information in peer reviewed published literature; presentations*); this part will help understand the current communication strategies and the drivers for using particular methods and procedures.
- *Social media, online and news media reporting*; depending on the setting and the country people use radio, microphone calling in the streets or other means to disseminate information. It is very important to understand what is used, where and by whom, so that the modified messages will successfully reach different groups of people.

### Development of vaccine safety messages

Baseline intelligence will be shared with the Research Committee following analysis by VSN members. Based on this analysis messaging will be customized to address concerns of different audiences and if required to be adapted to different events and specific needs of end-users. A manual (24) with GACVS guidance for vaccine safety communication on COVID-19 will be adapted to develop tailored messages for use on VSN members’ social media profiles, taking into consideration local languages, as well as sociocultural norms, beliefs and concerns of each setting and for other vaccines if needed. All campaign key messages will be developed by the Research Committee and VSN members in local languages and reviewed by a team of communication experts in collaboration with the WHO. Moreover, material and tools will adapted to follow the 5 steps guide of the cultural adaptation of health communication. Key messages will be provided to the VSN members for feedback and customization prior to implementation. Vaccine safety messages will be pre-tested by VSN members to obtain additional feedback from target audiences. We recognize that VSN members may be unable to split test messages due to limited resources. Therefore, further testing can be performed by the Research Committee upon request by VSN members before an immunization campaign or global event, such as HPV Awareness Day, World Immunization Week (WIW) or other health related event.

### Implementation, monitoring, and evaluation

The protocol will need to be submitted to the WHO Ethics Review Committee to seek clearance before performing the study. An implementation and evaluation guide will be supplied to VSN members, including data collection instructions. Data for this pilot will be collected using: (1) a post-communication campaign qualitative survey with target audiences; (2) social media monitoring; and (3) interviews with VSN members.

The post-campaign survey will provide insights into the knowledge, attitudes, and vaccine safety perceptions of target audiences following exposure to customized COVID-19 vaccine safety messages used by VSN members. Surveys will be designed by the Research Committee and will incorporate Larson et al. survey tool for measuring vaccine hesitancy with context specific adaptations (e.g., population, historical, socio-cultural values, politics) informed by VSN members (25). We will target a convenience sample of 150 men and women from each participating VSN member country. Recruitment strategy and inclusion criteria will be determined by VSN members. Meeting the convenience sample target will depend upon the outreach area and capacity of each VSN member. For example, a VSN member from Brazil may have fewer inhibitors to recruiting participants using social media than a member from Sudan where access to social media is more limited. Surveys and data collection will be administered by VSN members using a cloud-based platform. Data will be shared with the Research Committee for analysis and results will be shared with VSN members to discuss failure or success of messages and campaigns.

Additionally, social media monitoring of VSN member social media accounts such as Twitter or Facebook will be done by the Research Committee through social listening platforms. Key performance indicators (KPIs) (26) using a social media and web analytics curator will be monitored to provide additional data on impact of VSN messages and campaigns. Data such as conversion rate, reach, shares, likes, trends, retweets, comments, replies, and sentiments will be used to quantitatively assess messaging and implement changes to improve communication effectiveness and outreach. A social listening platform is currently available to all VSN members and provides the ability to measure social media performance across plugged social media channels and websites, as well as real-time content tracking and deep audience segmentation insights. For the purpose of the pilot study, a dashboard will be created to collect at the same time points as the public surveys KPIs, social media analytics data.

A list of candidate vaccine safety keywords will also be pooled in collaboration with VSN members. Candidate keywords will be selected based on language of VSN member countries and applied to a custom filter that will triage keywords for relevance. Keywords will be applied to Boolean searches in social listening platforms to collect additional data on vaccine safety social conversations beyond the campaign.

Interviewer-administered interviews with VSN members will be also performed by the Research Committee following the roll out of the vaccine safety communications. These interviews will be completed online. Interviews will take place over software that enables video/audio communication and recording. All interviews will be facilitated by a trained individual using a semi-structured interview guide. Participants will be deidentified by code name/study numbers. Transcripts of interviews will be recorded for analysis.

Qualitative data from the post-communication campaign survey will be coded using a deductive approach incorporating the expanded vaccine hesitancy conceptual model. VSN member interviews will be coded using an inductive approach. Thematic analyses will be performed by the Research Committee. Results of the surveys and interviews will complete the monitoring of messaging and will support the analysis of data received to better understand why communication was unsatisfactory or successful.

## Results

Most social media listening projects focus on collecting big data to identify and predict misinformation before they go viral. Messages are tracked using open source software or social listening platforms, and quantitative data is collected, aggregated, and analyzed over time (27). The primary outcomes of this study also involves the collection and analysis of social media KPIs on vaccine safety messages. We expect to obtain new knowledge on public attitudes and perceptions in LMICs as they are known to vary over time.

VSN members will also provide feedback on whether the pilot study process was feasible for them. Their feedback will provide new insight into their experience with the pilot project. For example, we will ask them if participant recruitment and data collection processes were burdensome. Another outcome of interest is whether they felt VSN educational resources on web and social media listening best practices adequately prepared them for the pilot study. Digital literacy and experience with social listening platforms may vary despite resources offered by the VSN. We also anticipate that not all VSN members have ample experience with pilot studies.

## Discussion

This pilot study is a unique and timely opportunity for the VSN to work with its LMICs members to improve and contribute to the ongoing development of vaccine safety communication standards. It will support understanding of the concerns and the important parameters to improve outreach of specific audiences. VSN members are highly valuable as they have local perspectives and insights into cultural, socio-economic, religious, and political factors that influence public perceptions on vaccine safety. Although the Network is heterogenous, it is a safe space gathering a lot of expertise and knowledge that favors collaboration establishment and mutual support. Collaboration with fellow VSN members that are fact checkers will enable less resourced VSN members to easily recognize misinformation that circulates not only from digital sources but local influencers. Through this study, VSN members can leverage all the advantages of global resources that facilitate social media surveillance, pharmacovigilance, and evidence-based guidance in the fight against the COVID-19 infodemic. New knowledge gained through this pilot study will be used to improve and further test the framework. Future applications of the framework will be explored to other communication campaigns aimed at promoting the safety of vaccines for routine vaccinations (e.g., measles, HPV, influenza, pneumococcus, polio, etc.).

### Ethics and dissemination

Concerns about the breach of personal privacy and confidentiality in the use of social media information as intelligence has been raised by experts (28). Social media analytics collected in this pilot study through the social listening platform as well as the interviews will be used in a broader analysis as methods to disseminate credible, accurate information on COVID-19 vaccination safety and changing personal views and behavior toward COVID-19 vaccination. Furthermore, social media data collected will only be sought from public spaces on platforms and stored on a password protect dashboard only accessible to the Research Committee. There will be no attempt to identify social media users and any information that can identify a user will be removed.

### Limitations

A possible limitation of this pilot study is the unintentional exclusion of target populations in LMICs that may have limited or no access to the Internet. Inclusivity will be an issue that we will address directly with VSN members. Mitigation strategies may include the use of alternate communication approaches to web sites and social media, such as radio ads, posters and pamphlets when required. Also, we will work with VSN members to ensure that local languages and terminologies for describing and communicating about vaccine safety are used in all aspects of this study.

The information collected will be used in a broader context involving the use of social media platforms as methods to disseminate credible, accurate information on vaccine safety not only for COVID-19 vaccine but also in view of new vaccines that are expected to be rolled out soon such as the Respiratory Syncytial Virus (RSV) or the Group B Streptococcus (GBS) vaccine in LMICs. Impact of this improved safety communication strategy can be measured by vaccine coverage before and after communication release and assess change in personal views and vaccine uptake. This information may enhance use of best practices in social media marketing by public health.

## Author contributions

LB contributed to conception and design of study and wrote the first draft of the manuscript. AT and FG contributed to conception and design of study. All authors contributed to the article and approved the submitted version.
